# Roles of virulence genes (*PsaA* and *CpsA*) on the invasion of *Streptococcus pneumoniae* into blood system

**DOI:** 10.1186/2047-783X-18-14

**Published:** 2013-05-17

**Authors:** Da-kang Hu, Dong-guo Wang, Yang Liu, Chi-bo Liu, Lian-hua Yu, Ying Qu, Xin-hua Luo, Jin-hong Yang, Jian Yu, Jin Zhang, Xiang-yang Li

**Affiliations:** 1 Department of Laboratory Medicine, Taizhou Municipal Hospital, 381 Zhongshan East Road, Jiaojiang District, Taizhou City, Zhejiang Province 318000, China

**Keywords:** *Streptococcus pneumonia*, Real-time PCR, Virulence gene, PsaA, CpsA

## Abstract

**Background:**

*Streptococcus pneumoniae* (SP) is the major cause of childhood mortality worldwide, we need to understand virulence genes of SP so can better target the treatment.

We investigated the expression of virulence genes *PsaA* and *CpsA* in different strains of SP interacting with monocyte cell line (THP-1) or pneumocyte cell line (A549) and the possible mechanism of SP invasion of the blood system.

**Methods:**

A total of 23 strains of SP were collected from hospitalized patients (blood-derived and sputum-derived) in the Second Affiliated Hospital of Wenzhou Medical College. The strains and ATCC 49619 were cultured, and RNAs were extracted. THP-1 and A549 cells were stimulated by different SP and ATCC 49619 for 4 h and 8 h, respectively. Quantitative real-time PCR was used to analyze the mRNA expression of *PsaA* and *CpsA*. The data were analyzed by SPSS 17.0.

**Results:**

The mRNA level of *PsaA* and *CpsA* were all significantly increased in clinical SP strains when compared to ATCC49619 after tedTHP-1 and A549 cells were stimulated. Clinical SPs showed higher virulence compared with ATCC49619.

**Conclusions:**

The expression of *CpsA* is the basis of the pathogenicity of SP. The expression of virulence gene *PsaA* may be helpful to the invasion of SP to the blood system.

## Background

*Streptococcus pneumoniae* (SP) is a class of the most common pathogens and is a leading cause of death for young children in developing countries. It is the pathogenic bacteria of community-acquired pneumonia, otitis media, meningitis, abscesses and so on, with infants and the elderly as the susceptible populations [1]. In developing countries, there are one million children younger than 5 years who die of pneumonia each year, and SP is one of the most fatal pathogens [2]. With high morbidity and mortality, SP is the most common pathogen present in the upper respiratory tract of asymptomatic carriers [3]; a variety of ingredients of SP such as capsule and other virulence factors could stimulate the immune response when SP changes from colonization to pathogenicity, especially during the process of blood or cerebrospinal fluid infection [4-6]; and various immune cells-such as neutrophils, monocytes/macrophages and dendritic cells-are involved in the process and release a variety of response factors simultaneously [7-9].

The proportion of septicemia and meningitis infections caused by SP accounts for about 5% in total SP infections, while the risk is much higher than other types with the fatality rate nearly exceeding 30% [10,11]. Some studies have reported on the mechanism of SP invasion of the blood system and nervous system; Uchiyama [6] reported that *NanA* was a virulence factor contributing to SP invasion of the cerebrospinal fluid (CSF). By now, more than ten kinds of virulence factors have been found in SP, such as *NanA*, *CpsA*, *CbpA, PsaA, PspA, PspC* and *Ply*, etcetera [2], each one has different bioactivity, and each can cause different types of SP infections [6,12,13].

Monocytes and pneumocytes are host defense cells in SP infections, and are commonly used for study of pathogenesis of SP infections [8,9]. In the current study, 23 clinical (blood-derived and sputum-derived) SP strains were isolated from hospitalized patients, and the representative SP virulence factors-*PsaA* and *CpsA*-were analyzed to explore the possible invasive mechanism of SP into blood system.

## Methods

### Bacterial strains and cell lines

SP standard strain ATCC49619 was provided by the Chinese National Center for Medical Culture Collections. Twenty-three clinical SP strains were isolated from the inpatients in the Second Affiliated Hospital, Wenzhou Medical College, in 2009. Written informed consent was obtained from each patient for the use of their clinical sample. The study was approved by the Institutional Ethnic Review Board of the Taizhou Municipal Hospital. SP infection diagnosis was confirmed by the hospital physicians based on the clinical symptoms and laboratory findings, whereas the polymerase chain reaction (PCR) test was used for the detection of *Pbp2B *[14] gene in SP.

A total of 23 non-duplicate strains were screened, 11 of them were isolated from specimens of blood, known as blood-derived SP and 12 strains were from sputum specimens, known as sputum-derived SP. All strains were identified to species by GPI Card of VITEK-32 automated microbial analyzer (bioMérieux Inc., Marcy-Etoile, France), and confirmed according to du Plessis [15] who reported an SP-specific PCR assay (target gene *Pbp2B*, forward primer: 5′-CTGACCATTGATTTGGCTTTCCAA-3′; reverse primer: 5′-TTTGCAATAGTTGCTACATACTG-3′, the length of purpose gene is 682 bp).

THP-1 and A549 cells were purchased from ATCC (Manassas, VA, USA). THP-1 cells were routinely maintained in RPMI 1640 medium, and A549 cells were maintained in F-12K medium, with 1% streptomycin, 1% penicillin and 10% fetal bovine serum (FBS) in a 5% CO^2^ incubator at 37°C. Both cell lines were adjusted to 3 × 10^8^/L for the experiment. 23 strains of SP were cultured and sublimated in normal saline at 1 and 2 Mcfarland for the cell stimulation test.

### Cell stimulation test

THP-1 cells were grown in 48-well plates with 10^5^ cells per well. A total of 100 μL of SP bacterial suspension with the concentration of 1.0 Mcfarland or 100 μL of normal saline as control was added into the plates. The cells were collected in 1.5 ml tubes by centrifugation (4000 rpm, 10 min) for RNA extraction immediately or stored in −80°C after culture in a 5% CO^2^ incubator at 37°C for 4 h or 8 h respectively. The method of the A549 cell stimulation test was the same as above.

### RNA extraction and real-time RT-PCR analysis

Total RNA was extracted with purified RNA-extracting reagent (Takara Biological Engineering Co., Ltd. Dalian, China). The purity of RNA was determined using a Nanodrop 2000 spectrophotometer, and OD260/OD280 values of the samples were approximately 1.8 to 2.2. Real-time RT-PCR was performed to detect transcripts of virulence gene (*PsaA* and *CpsA*), and the 16s rRNA of GAPDH was used as the endogenous control gene. The forward primer of *PsaA *[16] was 5′GGTACATTACTCGTTCTCTTTCTTTCT 3′ and the reverse was 5′GTGTGGGTCTTCTTTTCCTTTTTC3′; the forward primer of *CpsA* was 5′ AGTGGTAACTGCGTTAGTCC3′ and the reverse was 5′CTGCCAAGTAAGACGAACTC3′ [17]; and the forward primer of 16s rRNA was 5′GGTGAGTAACGCGTAGGTAA3′ and the reverse was 5′ACGATCCGAAAACCTTCTTC3′ [18]. PCR was performed with reaction mixtures containing 2.5 mM dNTP, 10 mM sense and antisense primers, and 5 units/ml TaqDNA polymerase in a thermal cycler for 30 seconds at 94°C, 30 seconds at 58°C (16 s rRNA), 56°C (*PsaA*), 65°C (*CpsA*), and 1 min at 72°C, 35 cycles; the final extension reaction was carried out at 72°C for 5 min.

### Statistical analysis

All data were analyzed by SPSS17.0 statistical software (IBM Corporation, NY, USA).

The *Kolmogorov-Smirnov* test was used for normal analysis. The *Levene* test was used for the two-sample homogeneity of variance test. One-sample *t* test was used for the comparison of the expression of virulence genes between clinical SP (including blood-derived SP and sputum-derived SP) and ATCC group. Factorial design analysis of variance was used for comparison between blood-derived SP and sputum-derived SP after stimulation. In addition, the two-sample *t* test was used for comparison of two groups before stimulation. The criterion of normality test was *P* < 0.10, which was also for the homogeneity of variance test. The criterion of significant difference of mean comparison was *P* < 0.05.

## Results

### The expression of virulence gene *PsaA* in *Streptococcus pneumoniae* was highly increased after THP-1 and A549 cells were stimulated

**Figure 1:**
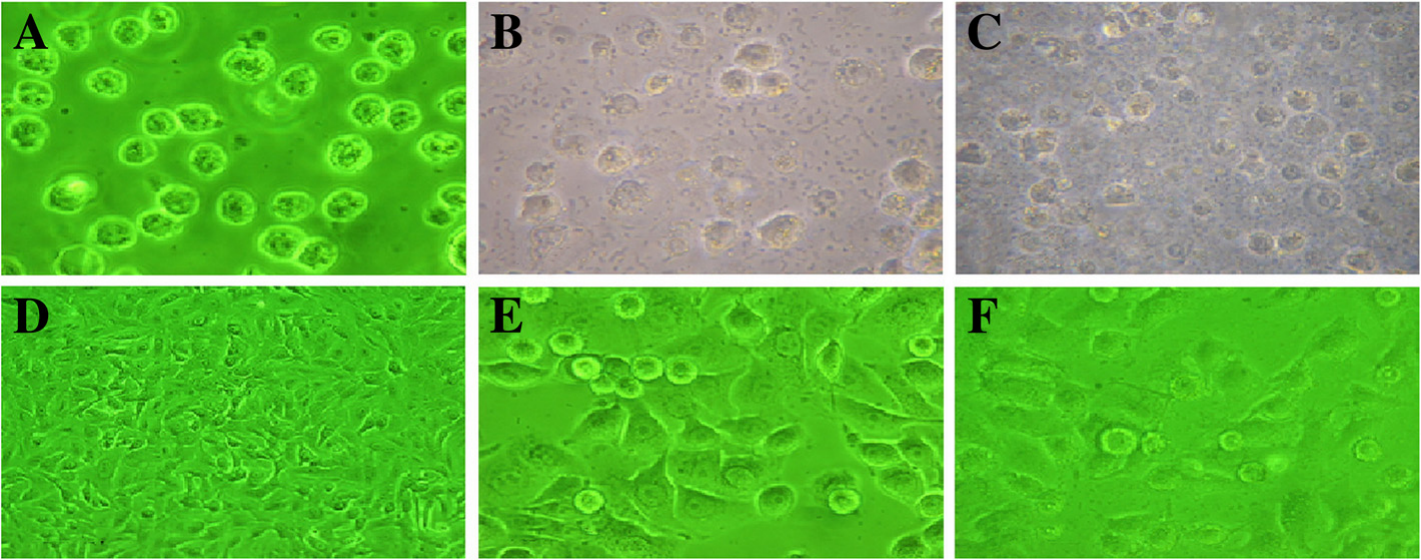
**Morphological changes of THP-1 and A549 cells after *Streptococcus pneumoniae *(SP) infection.** THP-1 and A549 cells began to die after infection at 4 to 8 hours: **A**) THP-1 at 0 hours; **B**) THP-1 at 4 hours; **C**) THP-1 at 8 hours; **D**) A549 at 0 hours; **E**) A549 at 4 hours; **F**), A549 at 8 hours. (×200).

The THP-1 and A549 cells were infected by SP, and we took pictures at 0 h, 4 h and 8 h to observe the morphological changes. SP was clearly observed 4 h after THP-1 and A549 were infected, and a large number of cells were dead after 8 h (Figure 1). The expression of virulence gene *PsaA* was detected by real-time RT-PCR after THP-1 and A549 cells were stimulated by SP for 0 h, 4 h, 8 h; all Ct values were normalized to the Ct of 16S rRNA and expressed as mean ± SD.

**Figure 2:**
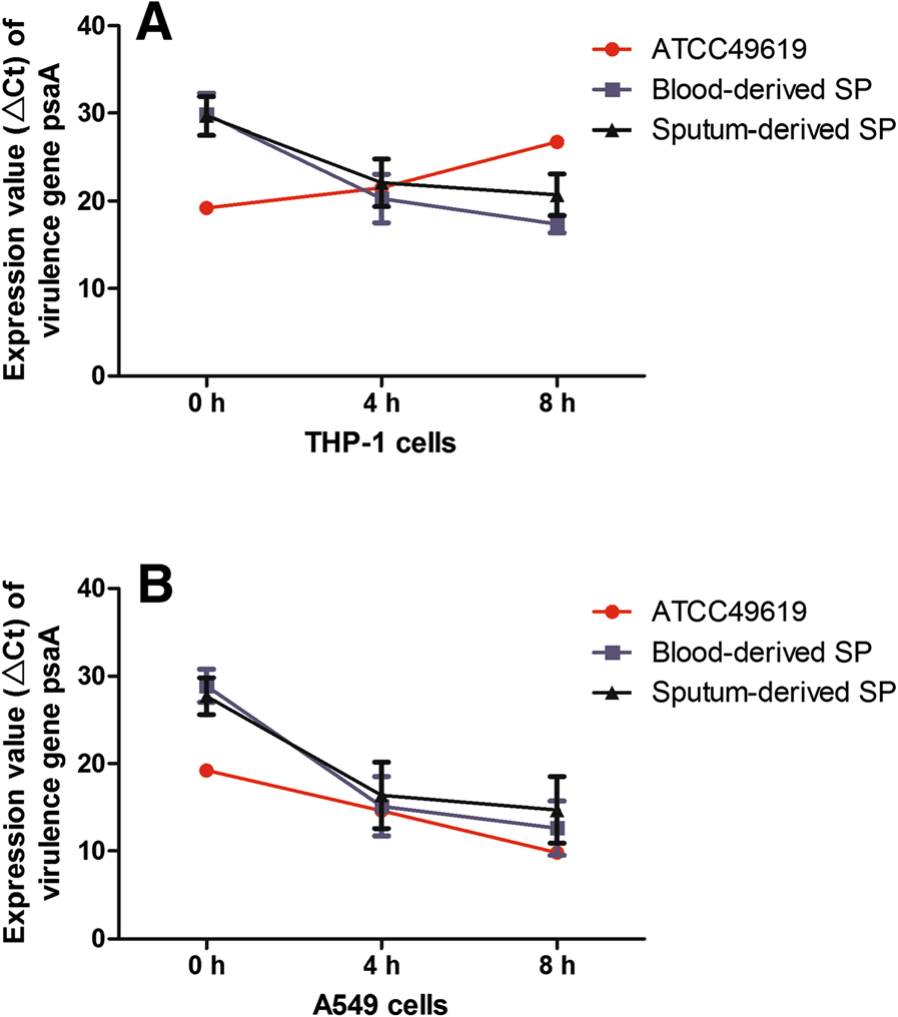
**The expression of *PsaA *gene in THP-1 (A) and A549 (B) cells at 0 hours, 4 hours, and 8 hours.**

For THP-1 cells, compared with the ATCC49619 group, the mRNA level of *PsaA* was lower initially in blood-derived and sputum-derived SP, but continued increasing after stimulation for 8 h. Virulence gene *PsaA* expression was increased in blood-derived and sputum-derived SP (ΔCt were 29.9 ± 2.4, 20.3 ± 2.8, 18.4 ± 1.0 in blood-derived SP group at the time points of 0, 4, 8 h, respectively, and 29.7 ± 2.2, 22.1 ± 2.7, 20.7 ± 2.4 in sputum-derived SP group at the time points of 0, 4, 8 h, respectively), while the expression of *PsaA* in ATCC 49619 strains was decreased (ΔCt increased from 19.2 at 0 h to 21.5 at 4 h and 26.8 at 8 h) (Figure 2A). At 8 h, *PsaA* mRNA level in blood-derived and sputum-derived SP groups was significantly elevated compared with ATCC 49619 strain (*P < 0.05*).

Factorial design analysis of variance was used for analysis of blood- and sputum-derived group comparisons. Results showed there was a significant difference (F = 5.066, *P* = 0.030) between blood- and sputum-derived group between 4 h and 8 h, while the interaction between the two groups and stimulation time point has no statistical significance (F = 0.111, *P* = 0.741).

In general, there was a significant difference of *PsaA* expression at 8 h when compared with expression at 4 h in clinically derived SP strains after THP-1 cells were stimulated; longer stimulating time further enhanced the SP *PsaA* expression, and when compared at the same stimulated time point, the expression of plasma-derived SP virulence gene *PsaA* was higher than the expression of the sputum-derived SP strains inTHP-1 cells. As we can see from Figure 2B, the expression of *PsaA* was increased in A540 cell after being stimulated by these three SP strains. The expression reached 19.2, 14.6, and 9.8 of ΔCt value for ATCC49619 strain after being stimulated for 0 h, 4 h and 8 h separately; the mean expression in blood-derived SP strains was 28.9 ± 1.9, 15.1 ± 3.4, and 12.6 ± 3.1 at 0 h, 4 h and 8 h separately; and the expression of sputum-derived SP strains was 27.7 ± 2.1, 16.4 ± 3.8, and 14.7 ± 3.8 at 0 h, 4 h and 8 h separately.

The *PsaA* expression level showed no significant difference at 4 h in these three groups, and the expression at 8 h showed a higher level of *PsaA* expressed in the blood-derived and sputum-derived SP groups compared with ATCC 49619 strain (*P* < 0.05) in A549 cells.

There was a significant difference (F = 4.132, *P* = 0.048) between blood- and sputum-derived groups between 4 h and 8 h, while the interaction between the two groups and stimulation time point showed no statistical significance (F = 0.176, *P* = 0.677).

In general, there was a significant difference of *PsaA* expression at 8 h when compared with expression at 0 h in clinically derived SP strains after A549 cells were stimulated; longer stimulating time further enhanced the SP *PsaA* expression; and when compared at the same stimulated time point, the expression of clinically derived SP virulence gene *PsaA* was lower than the expression of the ATCC49619 strain inA549 cells.

### The expression of virulence gene *CpsA* was highly increased after *Streptococcus pneumoniae* stimulated THP-1 and A549 cells

**Figure 3:**
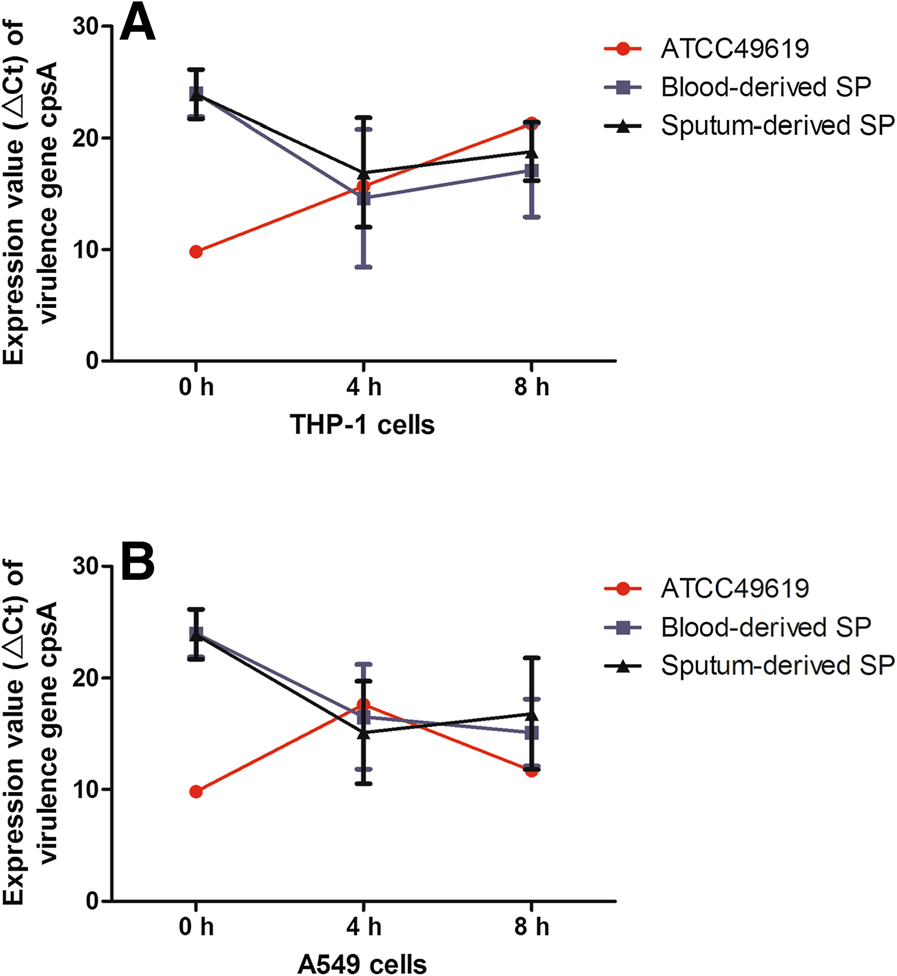
**The expression of *CpsA *gene in THP-1 (A) and A549 (B) cells at 0 hours, 4 hours, and 8 hours.**

Table 1 and Figure 3 showed the expression of the *CpsA* gene in these three strains after they stimulated THP-1 and A549 cells. For THP cells, the expression increased after clinically derived SP strains stimulated THP-1 for 4 h and decreased after 8 h; there was significant difference in the ΔCt values of the *CpsA* gene between clinical and ATCC49619 SP strains at 0 and 8 h (*P* < 0.05).

**Table 1:** **The expression of virulence gene *CpsA *of *Streptococcus pneumoniae *(SP) before and after stimulation of THP-1 and A549 cells**

**Group**	**Number**	**THP-1(ΔCt**^**a**^**, mean ± SD.)**			**A549(ΔCt, mean ± SD.)**		
		**0 hours**	**4 hours**	**8 hours**	**0 hours**	**4 hours**	**8 hours**
ATCC49619	1	9.8	11.7	25.3	9.8	17.6	11.7
Blood-derived SP	11	24.0 ± 2.1^b^	14.6 ± 6.2	17.1 ± 4.2^b^	24.0 ± 2.1^b^	16.5 ± 4.7	15.1 ± 3.0^b^
Sputum-derived SP	12	23.9 ± 2.2^b^	16.9 ± 4.9	18.8 ± 2.6^b^	23.9 ± 2.2^b^	15.1 ± 4.6	16.8 ± 5.0^b^

^a^Δ Ct = Mean Ct of tested gene - Mean Ct of control gene.

^b^*P* < 0.05, compared with ATCC49619 at same time point.

For THP1 cells, there was no significant difference between blood- and sputum-derived groups between 4 h and 8 h, and the interaction between the two groups and stimulation time point has no statistical significance as well.

For A549 cells, the expression of *CpsA* gradually increased after blood-derived SP stimulated A549 cells, and reached to the maximum at 8 h. The expression of *CpsA* was increased within 4 h, and then decreased slightly after sputum-derived SP stimulated A549 cells. There was significant difference in the ΔCt values of the *CpsA* gene between clinical and ATCC49619 SP strains at 0 and 8 h (*P* < 0.05).

For A549 cells, there was no significant difference between blood- and sputum-derived groups between 4 h and 8 h, and the interaction between the two groups and stimulation time point showed no statistical significance as well.

## Discussion

*SP* surface adhesion A (*PsaA*) is a lipoprotein on the surface of SP with the molecular weight of 37 kD [19]. It can adhere to the mucosal cells and other cells. With high homology to other streptococcal pilus, *PsaA* also can stimulate the body to generate protective antibodies to the infection of the lethal SP [20]. Our study showed that the expression of *PsaA* significantly increased in blood-derived and sputum-derived SP after they stimulated THP-1 cells, that the expression of *PsaA* was significantly different between the clinical SP and ATCC49619 groups, and that the *PsaA* expression was significantly higher in blood-derived SP group than that of sputum-derived SP group when THP-1 cells were stimulated for 8 h. These data indicate that the expression of *PsaA* in blood-derived SP was much more fatal than that of sputum-derived SP after infection for 8 h. The increased expression of *PsaA* contributed greatly to SP colonization, and it can work with other virulence factors to antagonize the stress of THP-1 cells. This may be one of the reasons that the blood-derived SP invades the blood system.

The expression of *PsaA* also increased in blood-derived and sputum-derived SP after A549 cells were stimulated, and the expression of *PsaA* was significantly different between clinical SP strains and ATCC49619. Same as THP-1, blood-derived SP strain showed a higher expression of virulence genes of *PsaA* than sputum-derived SP strains. Therefore, on the other hand, it also explained that elevated *PsaA* expression may be a factor in blood-derived SP invading the blood system.

The capsule is the most important virulence factor of SP, and more than 90 different serotypes have been found so far [21,22]. The importance of the SP capsule for virulence was demonstrated by the facts that (i) all the clinical isolates causing invasive disease are encapsulated; (ii) loss of the capsule by either genetic mutation or enzymatic degradation dramatically reduces virulence in animal models of infection [4,23-25]; (iii) different capsular serotypes vary in the ability to cause invasive diseases [26] and swapping capsular serotypes between strains affects virulence in animal models [27]; and (iv) opaque-phase variants (which expressed thicker capsule than transparent-phase variants) predominate during invasive infection [28]. The capsule can protect SP from complement-mediated phagocytosis and neutrophil clearance, which contribute to the occurrence and development of SP infection [29,30]. The C*ps* gene is responsible for the synthesis of SP capsular polysaccharide and the expression of *CpsA* was significantly different in this study between clinical SP and ATCC49619 after they stimulated THP-1 and A549 cells. The virulence gene *CpsA* expression levels were consistent between blood-derived and sputum-derived SP; this result demonstrated that the *CpsA* gene is necessary and provides the basis of pathogenesis for clinical SP.

ATCC49619 is a standard strain that has relative stability of physicochemical properties. Because of being isolated in external environment, it receives no influence from the external bacteria such as transformation and transduction, and when compared with clinical SP, ATCC49619 is a lower virulent strain with weaker survival ability.

## Conclusions

In general, the difference of virulence gene expressions between blood-derived and sputum-derived SP was demonstrated 8 h after THP-1 and A549 cells were stimulated. The results revealed that the alienation of virulence gene expression in different sources of clinical SP displays pressure to survive. Many virulence genes contribute to the invasion of SP; however, our study only demonstrates this for two of them-*PsaA* and *CpsA* genes. Thus, synergistic effect and correlation of these virulence factors is necessary for our future work.

## Competing interests

The authors declare that they have no competing interests.

## Authors’ contributions

DKH and XYL defined the research theme. DGW and YL designed methods and experiments, carried out the laboratory experiments, CBL and LHY analyzed the data, YQ and XHL interpreted the results and wrote the paper. JHY, JY and JZ co-worked on associated data collection and their interpretation. All authors read and approved the final manuscript.
